# The incidence of obesity, venous sinus stenosis and cerebral hyperaemia in children referred for MRI to rule out idiopathic intracranial hypertension at a tertiary referral hospital: a 10 year review

**DOI:** 10.1186/s12987-020-00221-4

**Published:** 2020-09-29

**Authors:** Grant Alexander Bateman, Gopinath Musuwadi Subramanian, Swee Leong Yap, Alexander Robert Bateman

**Affiliations:** 1 Department of Medical Imaging, John Hunter Hospital, Newcastle Region Mail Center, Locked Bag 1, Newcastle, NSW 2310 Australia; 2 Newcastle University Faculty of Health, Callaghan Campus, Newcastle, NSW Australia; 3 Paediatric Neurology, John Hunter Hospital, Newcastle, NSW Australia; 4 Biomedical Engineering, University of NSW, Sydney, NSW Australia

**Keywords:** Pseudotumor cerebri, Headache, Migraine, Venous sinus, Stenosis, Cerebral blood flow

## Abstract

**Background:**

Children referred to a tertiary hospital for the indication, “rule out idiopathic intracranial hypertension (IIH)” may have an increased risk of raised venous sinus pressure. An increase in sinus pressure could be due to obesity, venous outflow stenosis or cerebral hyperemia. The purpose of this paper is to define the incidence of each of these variables in these children.

**Methods:**

Following a data base review, 42 children between the ages of 3 and 15 years were found to have been referred over a 10 year period. The body mass index was assessed. The cross sectional areas and circumferences of the venous sinuses were measured at 4 levels to calculate the hydraulic and effective diameters. The arterial inflow, sagittal and straight sinus outflows were measured. Automatic cerebral volumetry allowed the brain volume and cerebral blood flow (CBF) to be calculated. The optic nerve sheath diameter was used as a surrogate marker of raised intracranial pressure (ICP). The sagittal sinus percentage venous return was used as a surrogate marker of elevated venous pressure. Age and sex matched control groups were used for comparison.

**Results:**

Compared to controls, the obesity rates were not significantly different in this cohort. Compared to controls, those at risk for IIH had a 17% reduction in transverse sinus and 14% reduction in sigmoid sinus effective cross sectional area (p = 0.005 and 0.0009). Compared to controls, the patients at risk for IIH had an arterial inflow increased by 34% (p < 0.0001) with a 9% larger brain volume (p = 0.02) giving an increase in CBF of 22% (p = 0.005). The sagittal and straight sinus venous return were reduced by 11% and 4% respectively (p < 0.0001 and 0.0009) suggesting raised venous sinus pressure. Forty five percent of the patients were classified as hyperemic and these had optic nerve sheath diameters 17% larger than controls (p < 0.0002) suggesting raised ICP.

**Conclusion:**

In children with the chronic headache/ IIH spectrum, the highest associations were with cerebral hyperemia and mild venous sinus stenosis. Obesity was not significantly different in this cohort. There is evidence to suggest hyperemia increases the venous sinus pressure and ICP.

## Background

Idiopathic intracranial hypertension (IIH) is characterized by increased intracranial pressure (ICP) in the absence of parenchymal brain lesions, vascular malformations, hydrocephalus or CNS infection [1]. To diagnose IIH, an elevated opening pressure above 28 cmH_2_O is required in children, unless they are not sedated or obese, then 25 cmH_2_O is the accepted level [2]. The ICP depends on several factors and is modeled using Davson’s equation:

1 $$ICP = FR_{CSF} x R_{out} + SSS_{p}$$

where *FR*_*csf*_ is the CSF formation rate, *R*_*out*_ is the CSF outflow resistance and *SSS*_*p*_ is the superior sagittal sinus pressure. The sagittal sinus pressure itself depends on Ohm’s law, i.e. the product of the outflow resistance and blood flow through the outflow plus the jugular bulb pressure [3]. So Eq. (1) can be expanded to:

2 $$ICP = FR_{CSF} x R_{out} + TCBF x R_{ven} + CVP$$

where *TCBF* is the total blood flow leaving the capillaries to enter the venous system, *R*_*ven*_ is the venous outflow resistance from the sagittal sinus to the jugular bulbs and *CVP* is the central venous pressure. Early theories regarding the pathogenesis of IIH in the literature have suggested an increase in the CSF formation rate, an increase in the CSF outflow resistance or an increase in the venous pressure [1]. However, the literature indicates the first two variables in both equations are found to be associated with active hydrocephalus rather than IIH. In children, CSF formation rates have been noted to be increased by 2–5 times in the hydrocephalus secondary to choroid plexus papilloma [4] and the CSF outflow resistance is increased in hydrocephalus secondary to acute hemorrhage and meningitis [5]. However, The CSF formation rate in adults is normal in IIH and decreases as the ICP increases [6]. Indeed, it has been found that the pressure gradient between the CSF and sagittal sinus is normal at 2.34 mmHg in adults with IIH [7]. Rearranging Eq. (1) by subtraction, the ICP to sagittal sinus pressure gradient is equal to the *FR*_*csf*_* x R*_*out*_, and if this term is normal, then the elevation in ICP in IIH can only be due to an elevated venous pressure. However paradoxically, it is noted children with an elevated venous pressure can present with either active hydrocephalus or IIH [8].

We can see from Eq. (2) that the venous pressure depends on the central venous pressure, venous outflow resistance and the total blood flow passing through the venous system. The central venous pressure in IIH has been found to be elevated due to obesity in adults [9]. The venous outflow resistance can be increased by venous sinus stenosis and the total cerebral blood flow can be increased in cerebral hyperemia [8].

Thus, the purpose of the current study is to define the incidence of obesity, venous sinus stenosis and/or cerebral hyperemia in a cohort of children referred for MRI imaging with the indication “rule out IIH” at a tertiary referral hospital over a 10 year period.

## Methods

### Subjects

An MRI with magnetic resonance venography (MRV) and blood flow quantification has been performed over a 10 year period as a comprehensive study into children with suspected IIH. The radiology information system at a tertiary referral hospital was retrospectively interrogated to retrieve all data from children between birth and 15 years of age who underwent an MRI with three dimensional T1 (3DT1), MRV and flow quantification, for the indication “rule out IIH” between September 2009 and September 2019. Forty two patients were found with ages between 3–15 years. Patients were excluded if there was evidence of hydrocephalus, active malignancy, active infection or thrombosis of the venous system. Ninety two control patients with MRV data between 3 and 15 years of age were enrolled from a previously published bank of patients undertaking MRI studies not related to headaches, large head or symptoms of raised intracranial pressure, in whom the subsequent MRI was found to be normal [8]. The control MRV patients consisted of 41 females and 51 males of average age 9.7 ± 3.9 years. From these controls 22 patients with flow quantification studies were selected. These controls consisted of 10 males and 12 females of average age 9.2 ± 4.1 years. The 42 patients at risk for IIH consisted of 20 males and 22 females of average age 9.5 ± 3.5 years. The ages were not significantly different between the groups. The clinical findings for the patients are listed in Additional file 1: Table S1. The blood pressure was recorded if it was measured within a month of the MRI study. The blood pressure was available in 64% of the blood flow controls and averaged 105 ± 12 mmHg systolic and 58 ± 12 mmHg diastolic. In the 42 patients at risk of IIH the blood pressure was available in 69% and averaged 100 ± 12 mmHg, 56 ± 13 mmHg and was not significantly different to controls. The body mass index (BMI) was calculated for each patient with the weight in kilograms being divided by the height in meters squared. In the 92 controls the average BMI was 18.6 ± 4.1, with 11 being overweight and 10 being obese when the BMI was reviewed with regards to age and sex specific cut off points [10], the average percentile score was 50 ± 34%. In those 42 patients at risk for IIH, the BMIs were larger than the controls at 20.5 ± 7.8 (p = 0.05), with 7 overweight and 10 obese, the average percentile score was 64 ± 14% being larger than the controls (p = 0.03). The fronto-occipital head circumference was measured. The average head circumference for the 22 controls was 51.9 ± 3.1 cm and in the patients at risk for IIH it was 53.6 ± 2.7 cm being 3% larger (p = 0.02). As is common in MRI of children, those aged between 3 and 7 years underwent a general anesthetic with both pulse oximetry and capnography monitoring. Conscious sedation is not utilized for children in our unit. The partial pressure of carbon dioxide was maintained between 35–40 mmHg by the anesthetist. There were no differences between the treatment of the controls and IIH patients.

### MR and analysis

**Fig. 1:**
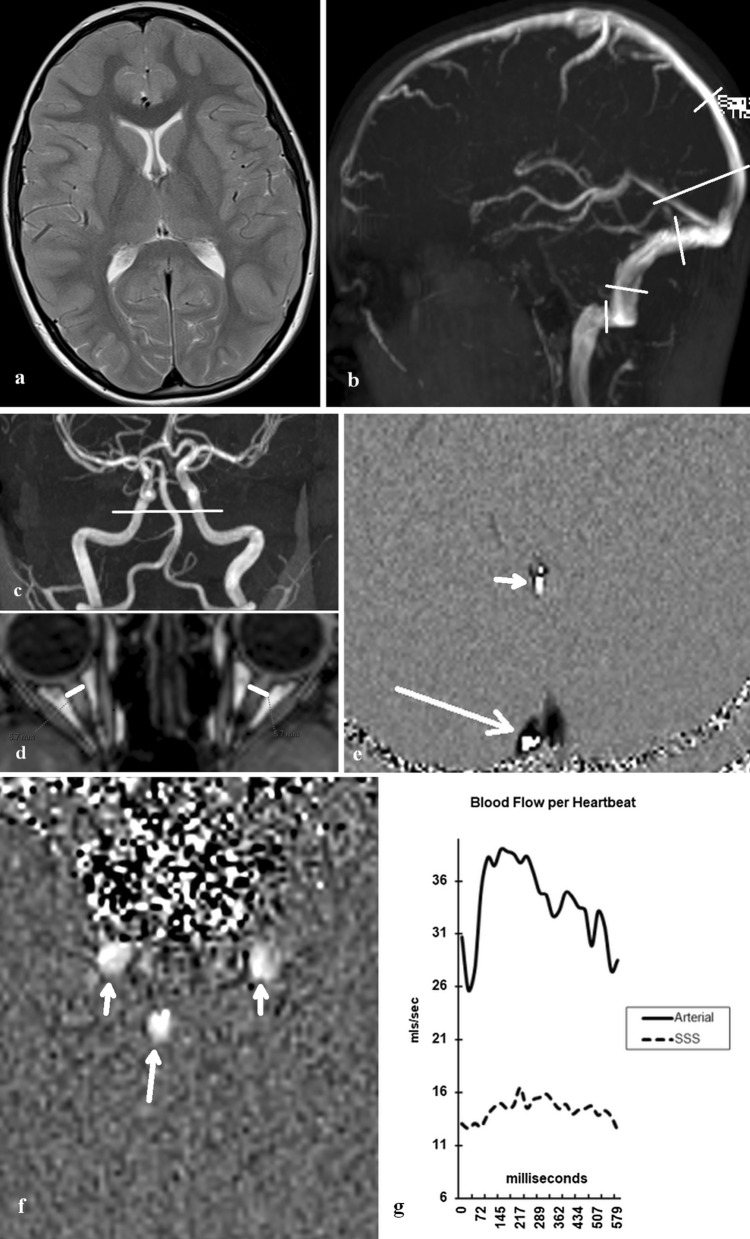
**a** A T2 sagittal image of patient 2, a child with chronic daily headaches and papilledema classified as not IIH showing no ventricular dilatation. **b** The MRV image for this patient with no outflow stenosis. The short lines show the site of the sinus area and circumference measurements. The large line shows the site of the venous flow quantification sequence. **c** An MR angiogram image of patient 2 with the line showing the site of the arterial acquisition for measuring arterial inflow. **d** A reconstruction of the 3DT1 data along the optic nerves with the lines showing the site of optic nerve sheath diameter measurement. **e** A phase image from the venous blood flow quantification acquisition in mid systole. The short arrow shows the straight sinus. The long arrow depicts the sagittal sinus. The apparent septum in the sagittal sinus is due to an early bifurcation of the sinus into the transverse sinuses which is a normal variant. The flow in the sinuses should be all black: the white areas indicate aliasing due to the peak flow rate being above 40 cm/sec so the baseline was changed from ± 40 cm/sec to − 51/ + 29 cm/sec. **f** A phase image from the arterial blood flow acquisition in mid systole. The short arrows indicate the two carotid arteries and the long arrow the basilar. There is no aliasing. **g** The flow results for the arterial and superior sagittal sinus flow. The arterial flow totaled 1960 ml/min with the sagittal sinus 770 ml/min or 39% of the inflow which is below normal

All patients were imaged on a 3.0 T superconducting magnet (Avanto; Seimens, Erlangen Germany). In all patients, a standard brain MRI consisting of 3DT1 sagittal (field of view 220 mm and matrix size 256 × 256), T2 axial, FLAIR axial and diffusion weighted axial images was performed, (see Fig. 1a). An MR phase contrast flow quantification sequence was acquired with retrospective cardiac gating. The TR was 26.5 ms, TE 6.9 ms, flip angle 15º, slice thickness 5 mm, matrix 192 × 512, FOV 150 and a single excitation. Two velocity encoding values were used. The first venc (velocity encoding) value was 40 cm/sec with the plane selected to pass through the sagittal sinus 3 cm above the torcular and through the mid part of the straight sinus (see Fig. 1b). The second venc value was 150 cm/sec with the plane set to pass through the skull base and cross the carotid and basilar arteries (see Fig. 1c). More information on the technique is available in a previous paper [3]. A time of flight MRV acquisition was performed in the off sagittal plane. This sequence does not require contrast and covered the entire head region (Fig. 1b). The MRI imaging was sourced from the hospital picture archiving and communication system (PACS) and therefore all measurements were performed on the original data.

Areas of interest were placed around the sagittal and straight sinuses to give the outflow for each, (see Fig. 1e). Areas of interest were placed around the carotid and basilar arteries for all patients, to give the total arterial inflow at the skull base by summing the individual flows (see Fig. 1f). Background subtraction was used to remove the effect of eddy currents. An arterial inflow of greater than 1410 ml/min was used to define cerebral hyperemia and a blood flow less than this figure defined normal blood flow. The percentage of the total blood inflow drained by each sinus was estimated by calculating the fraction each represented compared to the total inflow for each patient (see Fig. 1 g). The brain volume was calculated by anonymizing the 3DT1 data and uploading it to the Volbrain website [11]. The cerebral blood flow (CBF) was estimated in ml/100cm^3^/min by dividing the total arterial inflow by the brain volume. The MRV data was reformatted to display the cross-section of the sinuses at the midpoints of the distal half of the sagittal sinus, the transverse sinuses, the vertical segment of the sigmoid sinuses and the horizontal portion of the sigmoid sinuses as per the previous paper (see Fig. 1b) [8]. If an accessory occipital sinus was found this was also measured. At each site, the cross-sectional area of the sinus and the wetted circumference was measured using the scanners measurement tool. The hydraulic diameter of each cross-section was calculated using the formula:

3 $$Hd = {\raise0.7ex\hbox{${4A}$} \!\mathord{\left/ {\vphantom {{4A} {Circ}}}\right.\kern-\nulldelimiterspace} \!\lower0.7ex\hbox{${Circ}$}}$$

where *Hd* is the hydraulic diameter, *A* is the cross-sectional area of the sinus and *Circ* is the wetted circumference of the sinus. In the areas below the Torcular, there was more than one parallel pathway for blood to flow, the effective diameter of each segment was calculated using the formula:

4 $$D_{e} = \left( {\Sigma D_{i}^{{{\raise0.7ex\hbox{$5$} \!\mathord{\left/ {\vphantom {5 n}}\right.\kern-\nulldelimiterspace} \!\lower0.7ex\hbox{$n$}}}} } \right)^{{{\raise0.7ex\hbox{$n$} \!\mathord{\left/ {\vphantom {n 5}}\right.\kern-\nulldelimiterspace} \!\lower0.7ex\hbox{$5$}}}}$$

where *D*_*e*_ is the effective diameter, *D*_*i*_ is the hydraulic diameter of each parallel sinus segment and *n* is the number of parallel segments. A hemodynamically significant stenosis was taken to be a reduction in effective area of 65% or greater with a non-significant stenosis being between 50 and 65% [8]. A search was made in both the control and patient groups for non-significant or significant stenoses.

The optic nerve sheath diameter was measured 3 mm behind the globe by reconstructing the 3D T1 data along the nerve sheaths (see Fig. 1d). The right and left measurements were averaged for each patient. Evidence of optic globe flattening or pituitary compression (empty sella) was obtained from the planar imaging. Mean and standard deviations were obtained for each group. A Shapiro–Wilk Test was used to test for normality of the data. A Chi square test with p value of 0.05 was used to test for significance in the non-continuous data. Differences between continuous data groups were tested using a non-paired t-test with a confidence level of 0.05 set for the majority of measurements and a level of 0.0125 for the stenosis measurements following Bonferroni correction to reduce the possibility of family-wise error. Correlation amongst continuous variables was performed using a Spearman’s Rho test.

## Results

The average sinus size findings are summarized in Table1 and the average optic nerve sheath diameters and blood flow findings for controls, those with an arterial inflow above 1410 ml/min (hyperemic), those below this cut-off (non-hyperemic) and the entire at risk cohort are summarized in Table 2. The raw data for these tables is available in the Additional file 1: Tables S1 and S2.

**Table 1:** Venous sinus measurements in control group and those at risk for IIH

	Age years	SSS Hd mm	TS Ed mm	SS Ed mm	DSS Ed mm
Control					
Mean	9.7	6.8	8.1	8.3	8.0
SD	3.9	0.9	1.1	1.4	1.5
n = 92					
At risk for IIH					
Mean	9.5	6.5	7.4	7.7	8.2
SD	3.9	0.9	1.3	1.0	1.8
n = 42					
t-test	0.82	0.07	0.005*	0.0009*	0.71

*DSS* distal sigmoid sinus, *Ed* effective diameter, *Hd* hydraulic diameter, *mm* millimeters, *SD* standard deviation, *SSS* superior sagittal sinus, *SS* sigmoid sinus, *TS* transverse sinus; *, significance < 0.05

**Table 2:** Mean cerebral blood flow and optic nerve sheath diameter

Case	Age years	Arterial inflow ml/min	Sagittal sinus flow ml/min	Straight sinus flow ml/min	SSS % flow	ST % Flow	Brain volume cm^3^	CBF ml/100cm^3^/min	Average ONSD mm
Control									
Mean	9.2	990	560	170	57	17	1224	82	5.3
SD	4.1	210	130	70	8	5	219	15	0.6
n = 22									
At risk for IIH Hyperemic									
Mean	8.3	1620	670	190	42	12	1358	120	6.2
SD	3.5	190	150	60	10	3	1136	16	0.9
n = 19									
t-test	0.48	< 0.0001*	0.01*	0.26	< 0.0001*	0.001*	0.03*	< 0.0001*	0.0002*
At risk for IIH non Hyperemic									
Mean	10.5	1010	530	140	48	13	1321	84	5.1
SD	3.4	190	110	60	7	5	152	16	0.7
n = 23									
t-test	0.25	0.09	0.41	0.23	0.001*	0.02*	0.12	0.58	0.26
All cohort									
Mean	9.5	1330	600	170	46	13	1334	100	5.6
SD	3.5	310	150	60	10	4	145	24	1.0
n = 42									
t-test	0.73	< 0.0001*	0.34	0.88	< 0.0001*	0.0009*	0.02*	0.005*	0.19

*cm*^*3*^ centimeter cubed, *SSS* superior sagittal sinus, *ST* straight sinus, *ml/100cm*^*3*^*/min* milliliters per 100 cm cubed per minute, *mm* millimeters, *mm*^*2*^ millimeters squared, *SD* standard deviation, *ONSD* optic nerve sheath diameter; ^*^, t-test p value < 0.05. All t-tests compare patient groups with the control group

In the control MRV group, 30% of systems showed one side to be dominant i.e. the contralateral side was smaller by 50% or greater in area compared to the average with 70% being codominant. In the controls no significant effective diameter sinus stenosis was seen but 5 non-significant stenoses were seen for a total stenosis rate of 5%. In the 22 controls where the optic nerve sheath was measured the diameters averaged 5.3 ± 0.6 mm. There was no orbital flattening or evidence of empty sella.

The patients at risk for IIH showed a 25% unilateral dominant sinus rate and 75% codominant which was not significantly different to controls. Compared to the 92 MRV controls, the 42 patients at risk for IIH had a 17% reduction in transverse sinus and 14% reduction in sigmoid sinus effective cross sectional area (p = 0.005 and 0.0009) there was no significant difference in the sagittal and distal sigmoid sinuses (Table 1). There were no focal stenoses in the sagittal sinuses but 3 patients had a focal transverse sinus stenosis (71% stenosis for case 8, 60% for case 13 and 62% for case 38) and 3 patients had stenoses in the sigmoid sinus (64% stenosis for case 3, 51% for case 12 and 55% for case 21) for a total stenosis rate of 14% which was not significantly different to controls (p = 0.08). In the patients at risk for IIH, the total arterial inflow in ml/min was 34% larger than the 22 controls (p < 0.0001) but the sagittal and straight sinus outflows were not significantly different to controls (Table 2). The percentage of the arterial inflow returning by each sinus was 11% smaller for the sagittal sinus and 4% smaller for the straight sinus compared to controls (p = 0.0001 and 0.0009).The brain volumes were 9% larger than the controls (p = 0.02), giving a 22% increase in CBF in ml/100 cm^3^/min (p = 0.005). The optic nerve sheath diameters were 5.6 ± 1.0 mm, being not significantly different to controls. There were 10 cases with orbital flattening (cases 3, 5, 8, 10, 11,18,19,21, 23 and 32) and 4 with a partial empty sella (case 8, 20, 23 and 32). There were 19/42 or 45% of the 42 patients at risk for IIH who had arterial inflows greater than two standard deviations above the mean for the controls. Compared to controls these hyperemic patients showed sagittal sinus and straight sinus venous returns reduced by 15% and 5% (p < 0.0001 and = 0.001 respectively), the CBF was increased by 46% (p < 0.0001) and the optic nerve sheath diameters were increased by 17% (p < 0.0001, Table 2).

**Fig. 2:**
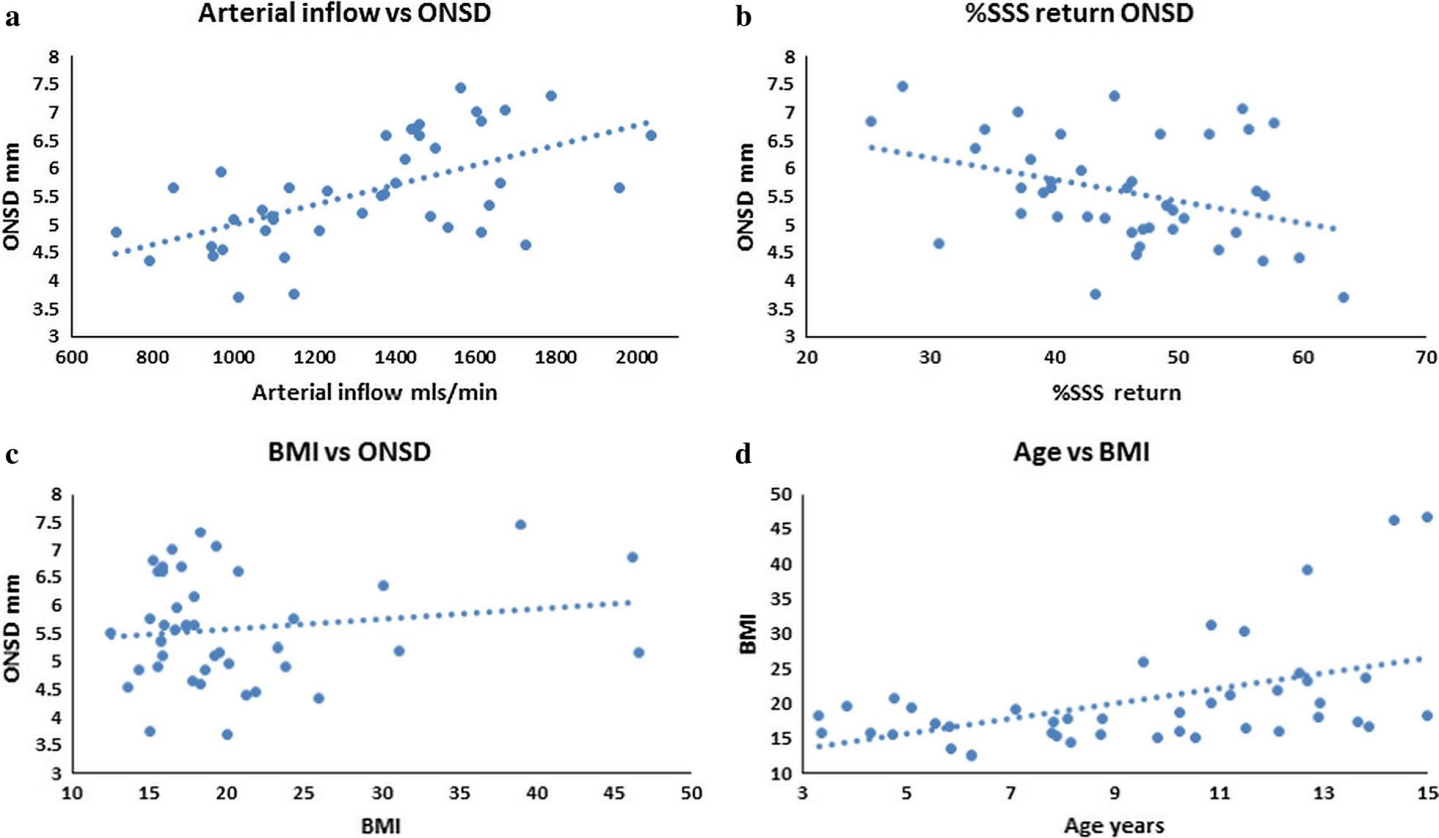
**a** A Scatter plot of arterial inflow vs optic nerve sheath diameter (ONSD) showing a moderate positive correlation (r = 0.58, p < 0.0001) suggesting higher arterial inflow increases ICP. **b** A Scatter plot of the percentage sagittal sinus (SSS) return vs optic nerve sheath diameter (ONSD) with a weak negative correlation (r = − 0.35, p = 0.02) suggesting a reduced venous return correlates with an elevation in ICP. **c** A Scatter plot of body mass index (BMI) vs optic nerve sheath diameter (ONSD) showing no overall correlation. **d** A Scatter plot of Age vs body mass index (BMI) showing a moderate positive correlation (r = 0.47, p = 0.002) suggesting an increase in obesity related venous pressure may become more important with age

In the 42 patients there was a moderate positive correlation between age and BMI (r = 0.47, p = 0.002) and arterial inflow and ONSD (r = 0.58, p < 0.0001) (Fig. 2). There was a weak negative correlation between age and arterial inflow (r = − 0.37, p = 0.02), between arterial inflow and %SSS return (r = − 0.38, p = 0.01) and between %SSS return and ONSD (r = − 0.35, p = 0.02) (Fig. 2). There was no correlation between any other variable.

## Discussion

Using the revised criteria for IIH in children a lumbar puncture (L.P.) is always required [2]. An elevated opening pressure above 28 cmH_2_O is specified, unless the patient is not sedated or obese then 25 cmH_2_O is the accepted level [2]. Unfortunately, L.P. is an invasive procedure and is often difficult to interpret in a distressed child. This mandates the use of heavy sedation or a general anesthetic which is often not practical or without risk. Thus only five of the children in our cohort underwent L.P. This makes the exact classification of children difficult using the available criteria. According to the revised criteria, “definite” IIH requires raised ICP and either papilledema or abducens nerve palsy. “Probable” IIH has a normal CSF pressure in the presence of papilledema and “suggestive” IIH has raised ICP without papilledema, but 3 out of 4 additional imaging criteria i.e. empty sella, flattening of the posterior globe, distention of the optic nerve sheath or transverse sinus stenosis. According to these criteria, 2 of the patients have definite IIH and one probable. There were 6 patients with headache plus papilledema who did not have an L.P. and therefore could not be classified. We have used optic nerve sheath dilatation as a surrogate marker of raised ICP and compared this to two markers of altered blood flow i.e. cerebral hyperemia (where the arterial inflow is greater than two standard deviations above the mean) and a sagittal sinus return less than 41% (the percentage is compared to the arterial inflow with 41% being less than two standard deviations below the mean). In the 6 patients with papilledema and headache, (despite failing formal classification) 4 had optic nerve sheath dilatation, 4 had a sagittal sinus venous return below 41% and 4 had arterial hyperaemia, suggesting an elevated ICP and venous pressure. The findings in these 6 children indicate the definition of “suggestive IIH” may be too stringent to be clinically useful in young children. The remaining 31 patients (who also failed to meet the criteria for “suggestive” IIH) have, in the majority, chronic daily headaches suggestive of transformed migraine. However, of these later patients, 7/31 have optic nerve sheath dilatation and 8/31 had a sagittal sinus return below 41%, suggesting some component of raised ICP and raised venous pressure. The findings suggest a continuum in ICP may exist from definite or probable IIH to those with headache plus papilledema to those without enough evidence to suggest IIH but having chronic headaches. De Simone et al. suggested that a continuum exists between IIH, IIH without papilledema (IIHWOP) and chronic migraine [12]. The clinical presentation of IIHWOP may be indistinguishable from chronic migraine [13] and 14% of patients with refractory transformed migraine had IIHWOP at lumbar puncture [14]

### Normal controls

The 22 blood flow controls were selected from patients undergoing MRI diagnostic studies for indications excluding enlarged head, raised ICP, hydrocephalus or headaches. The indications were principally seizures, ear disorders or pituitary disease, where the MRI was interpreted as normal [8]. The average head circumference was 51.9 ± 3.1 cm with the average brain volume being 1224 ± 219 cm^3^. At autopsy, the average brain weight for 8–9 year old females is 1180 gm and males 1370 g [15]. As

12/22 subjects were female, the average brain volume is similar to the autopsy series brain weight given a normal brain density is 1.04 g/cm^3^ [16]. The sinus size measurements utilised a technique to standardise the diameters of each triangular or oval sinus to an equivalent cylindrical tube (hydraulic diameter) and then collapse the multiple parallel pathways below the torcular into a single equivalent tube (effective diameter) to make direct comparisons between patients more robust (see [8] for further details). Under these conditions 5% of control children had an effective focal stenosis which would increase the venous pressure by between 2 and 5 mmHg [8].

**Fig. 3:**
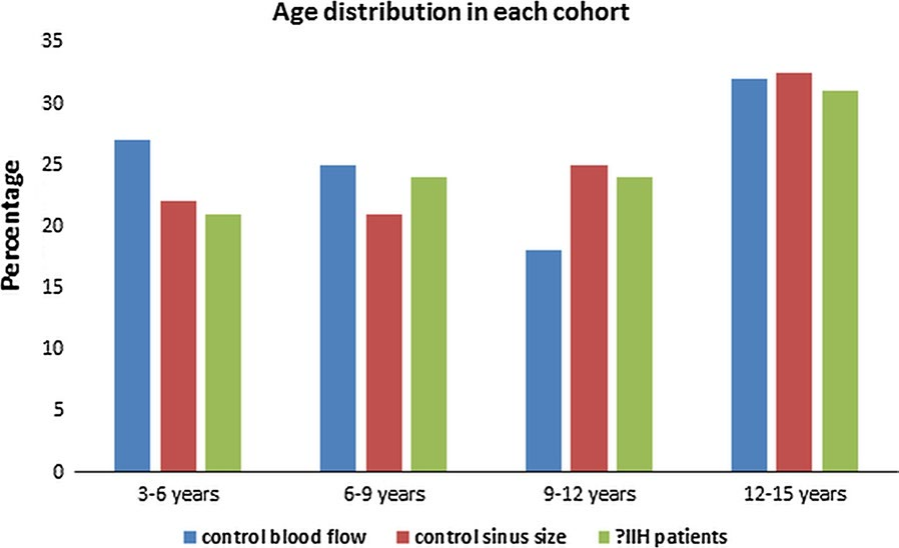
A histogram showing the age distribution for the two control groups vs the patients showing no significant differences

In children, the cerebral blood inflow varies with age, peaking at about 8 years of age and then reducing over the course of development [17]. The average arterial inflow of 990 ± 210 ml/min gave an upper limit of normal i.e. 2 standard deviations above the mean of 1410 ml/min. This is the cut-off used to define hyperemia. By this definition, no control child had hyperemia. Given the arterial inflow and brain volume, the controls had an average CBF of 82 ± 15 ml/100 cm^3^/min. Using SPECT 133Xe, Chiron et al. found the CBF peaked between 4 and 8 years at 71 ml/100 g/min [18] which is similar to our findings. In a study using arterial spin labelling of the whole brain, children of average age 8.1 years had a CBF of 83 ± 19 ml/100 g/min [19], which is also similar to our findings. We found a weak negative correlation between blood flow and age with the age distribution between controls and patients being not significantly different (see Fig. 3). The percentage of the arterial inflow returning through the sagittal sinus was 57 ± 8%, giving a lower limit of normal (2 standard deviations below the mean) of 41%, the straight sinus % return was 17 ± 5%. No control child had a venous return for the sagittal sinus below 41%. There was no secondary evidence of raised ICP in the controls with no evidence of empty sella or globe flattening. The optic nerve sheathes averaged 5.3 ± 0.6 mm. In a published study, when the optic nerve sheath diameters were measured 3 mm behind the globe using MRI, in children older than 1 year of age, the normal diameter was found to be 5.24 ± 0.42 mm (similar to our series) and in those with increased ICP the diameter averaged 6.38 ± 0.78 mm. A threshold of 6 mm gave a 71% sensitivity and 90% specificity for an elevated ICP [20].

### Obesity and venous sinus stenosis in patients at risk for IIH

It has been said that an elevated venous pressure is the universal mechanism underlying IIH [21]. The cerebral venous outflow pressure depends on Ohm’s law, i.e. the pressure equals the product of the outflow resistance, blood flow through the outflow plus the jugular bulb pressure [3, 8]. In adults with IIH, 71% are obese [22]. Obesity raises the central venous pressure by up to 20 mmHg [9, 23] (the third component of Ohm’s law) and therefore sinus pressure in IIH. In comparison, in a large series of children with IIH, in those less than 12.4 years of age, 45% were obese [24]. In the current study, both patients with definite IIH were obese, the probable IIH patient was not (Additional file 1: Table S1). In the patients who failed the diagnostic criteria for IIH, 8/39 or 21% were obese which was not significantly different to the controls (p = 0.33). There was a positive correlation between age and BMI suggesting obesity may become more important with age (See Fig. 2).

Up to 90% of adults with IIH have been found to have an outflow stenosis (the first component of Ohm’s law) raising the venous resistance and therefore the venous pressure [12]. The stenoses tend to be of high grade, with pressure gradients ranging between 4 and 41 mmHg in one series [25]. It can be seen that there must be a considerable overlap in adults with IIH with up to 2/3 having both obesity and stenosis. Ahmed et al. found the sagittal sinus pressure to be increased by 26.5 mmHg [25] above the normal figure of 7.5 mmHg [26]. The pressure drop across the venous system averaged 20 mmHg [25] compared to the normal figure of 2.5 mmHg [26]. Thus by subtraction, the jugular bulb pressure in the Ahmed et al. [26] study was 14 mmHg compared to the normal value of 5 mmHg. From this we can deduce that in adults with IIH, 66% of the increase in ICP is due to the venous stenosis and 34% is due to obesity elevating the central venous pressure. Thus the great majority of adults with IIH can be explained on the basis of one or both of these two variables operating. In a review of children of average age 9 years with IIH, only 13% showed evidence of venous sinus stenosis [27]. In another study of 145 pediatric IIH cases, 52% showed dominant-side venous narrowing [28]. In the current study one out of the three patients with definite or probable IIH had a focal stenosis of 71%. Both of the definite IIH cases were also obese. Therefore, it is likely there is overlap, with both variables occurring in some children similar to adults. If there were a 50% overlap, then at best, only 70% of pediatric cases in the literature could be explained on the basis of obesity ± stenosis. Therefore, if an elevation in venous pressure is the underlying cause of all childhood IIH, then at least 30% of cases in the literature would require an elevation in CBF (the second component of Ohm’s law) for this to occur. The non IIH focal stenosis rate was not significantly different compared to the controls.

### Blood flow in headache and IIH

The MR flow quantification sequence used in this study is a standard acquisition protocol and is available on most commercial scanners. The sequence works by measuring the phase angle change induced in the spins of the protons as they pass through the magnetic field of the scanner in the slice selected. The phase change is proportional to the velocity provided the selected velocity encoding or Venc value is correct. As there are only + 180 and − 180 degrees of phase change available to detect flow, the maximal flow velocity expected must be selected. If too low a Venc is selected then velocities faster than this rate will be aliased and depicted as being in the opposite direction (see Fig. 1e). Aliasing can be reversed by altering the velocity encoding to allow a greater range in the aliased direction at the expense of reducing the range in the opposite direction. The sequence is gated to the heartbeat and approximately 200 heartbeats are combined, giving, on average, 24 segmented measurements of velocity per R-to-R interval depending on the heart rate and the TR. As the velocity across the entire heartbeat can be averaged for a region of interest, the volume passing through this region can be measured by multiplying the velocity obtained by the area of the region. Eddy currents induced in the slice can give an error in the measurement which is eliminated by subtracting this phase offset obtained from an adjacent non-moving area of interest [29]. This technique has been validated both in vitro and in vivo and found to have error rates in the order of 5%, with a tendency to slight underestimation of flow; the intra- and inter-observer variability is described as low to negligible [30–32].

Hyperemia is defined as an increase in the amount of blood in a part, organ or tissue as a result of dilatation of the supplying arteries [33]. In adults who have IIH but no evidence of venous outflow stenosis, an increase in the arterial inflow of 46% has been noted [34], however because up to 90% of adults have venous stenoses, hyperemia in adults is probably rare. In contrast, we have found it is relatively common for children at risk for IIH to have arterial inflows greater than two standard deviations above the mean with 45% so affected (Table 2). In this cohort, both of the definite IIH patients have an elevated CBF, the probable IIH patient did not. Meaning that 17/39 or 44% of those classified as not having IIH had an elevated blood flow. Mathematical modelling has shown that elevated venous pressure and IIH can occur either due to a significant venous stenosis and normal arterial inflow or an elevated inflow with a small component of venous buckling which would be difficult to see with MRV [35]. In the current cohort, the inflow is elevated by 34% and there is buckling of the transverse and sigmoid sinuses with a 17 and 14% reduction in cross-section respectively. An elevation in venous pressure secondary to an increased arterial inflow is not a new finding. Increasing the partial pressure of carbon dioxide in the lungs increases the CBF. Global CBF increases by 1–2 ml/100 g/min for each 1 mmHg change in Paco_2_ [36]. In a primate model, an increase in Pa_CO2_ between 60 and 80 mmHg increased the venous outflow pressure by 65% [37]. Finally, increasing the venous pressure increases ICP. Shawcross et al. [38] found a 17% increase in cerebral blood flow led to a 33% increase in intracranial pressure.

### Diagnosing IIH in children

**Fig. 4:**
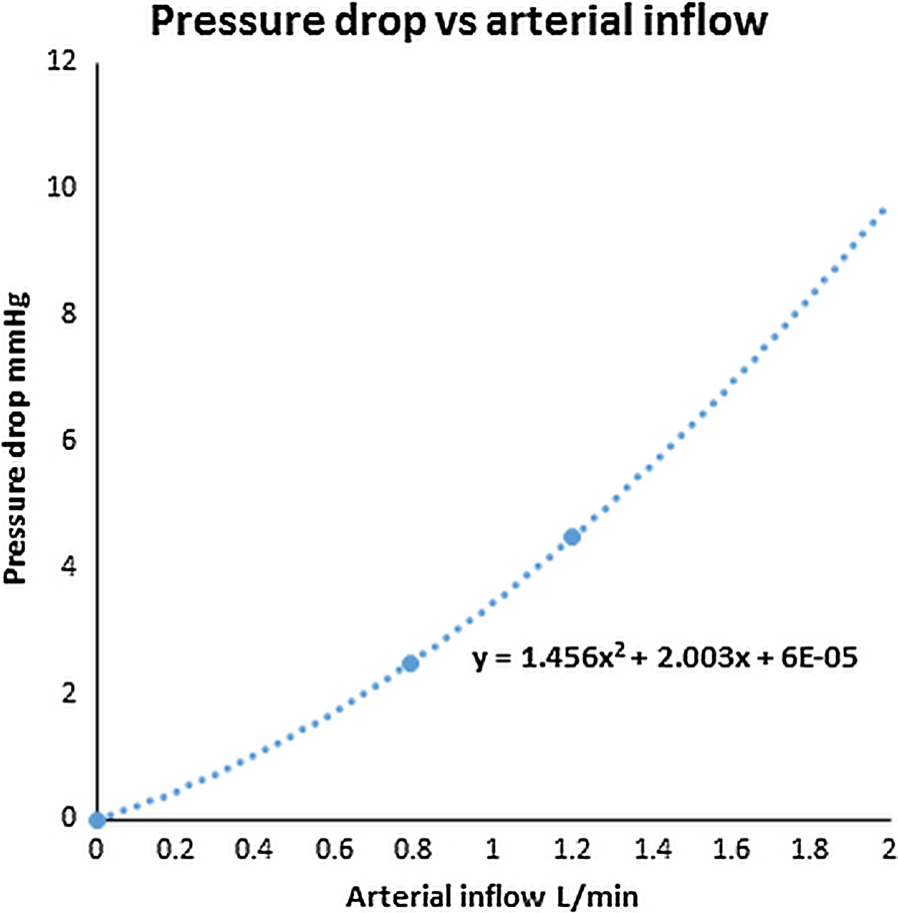
A plot of pressure drop across the venous system vs arterial inflow based on three points from the literature with extrapolation. The curve of best fit is quadratic and not linear

**Fig. 5:**
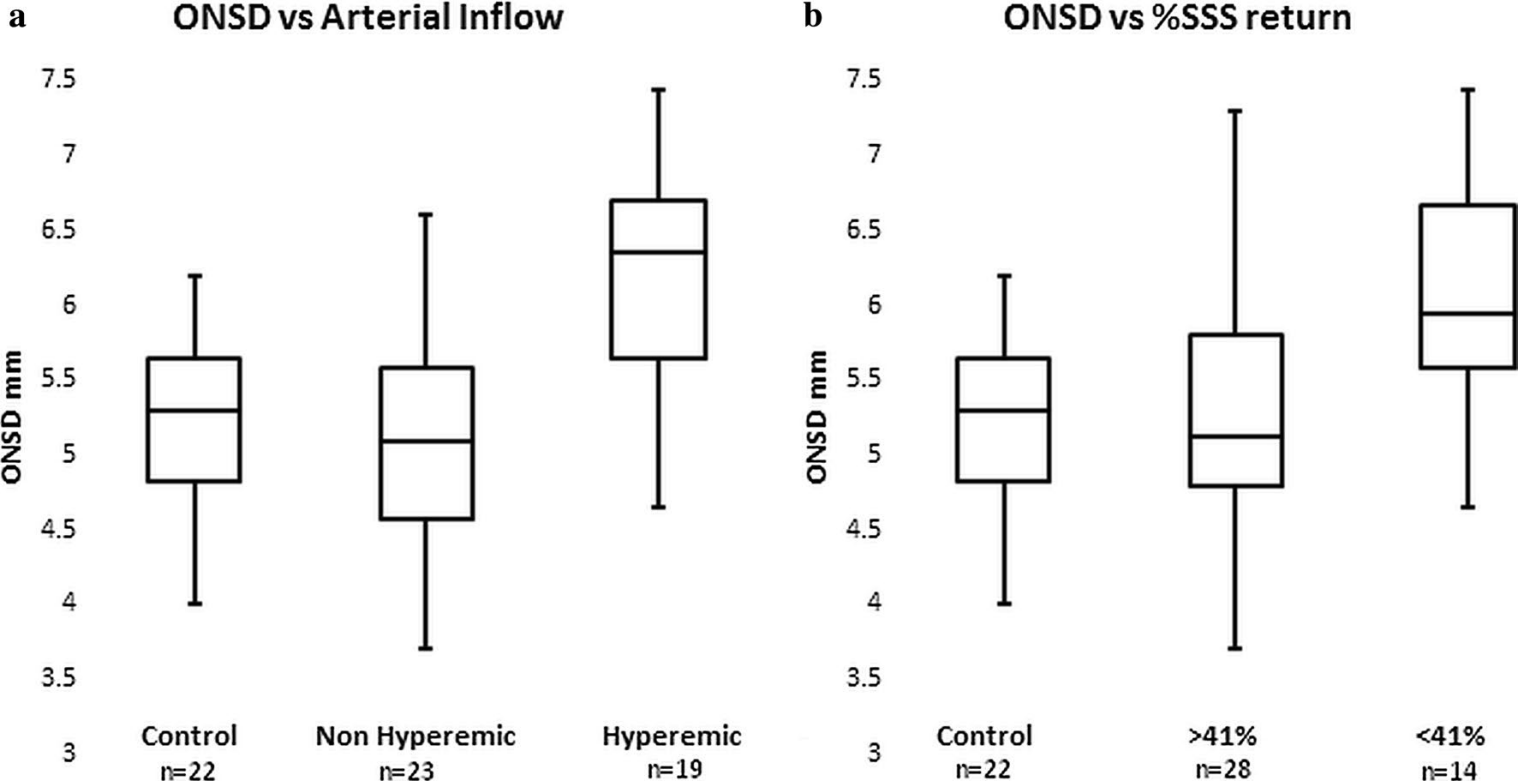
**a** A box and whisker plot of the optic nerve sheath diameter (ONSD) in controls and patients at risk of IIH who had arterial inflow both above and below 1410 ml/min. Hyperemia correlates with optic nerve sheath dilatation and therefore elevated intracranial pressure. **b** A box and whisker plot of the optic nerve sheath diameter (ONSD) in controls and patients at risk of IIH who had sagittal sinus percentage (%SSS) returns both above and below 41%. Reduced venous return correlates with optic nerve sheath dilatation and therefore elevated intracranial pressure

The normal ICP in children is 14.6 mmHg [39], meaning the revised criteria require the ICP and the sinus pressure to be increased by 3.8 mmHg in most children or 6 mmHg in an obese or sedated child. From Ahmed et al. [8], (as discussed above) obesity in adults can elevate the ICP by an average 9 mmHg meaning morbid obesity on its own could trigger IIH in children. Similarly, a 65% stenosis can elevate the venous pressure and ICP by 10 mmHg and also trigger IIH on its own in children. As noted in the paper by Shawcross et al. [38] (discussed above) the response of the ICP to blood flow is non-linear with a 17% increase in CBF giving a 33% increase in ICP and this warrants further consideration. Given there is no correlation between age and sinus size in the 92 controls (i.e. after 3 years old the sinus size is fully developed), three points on the pressure vs volume curve for a normal control can be derived from the literature. Point one, if there is no flow, the pressure drop from the sagittal sinus to jugular bulb is zero. Point two, in adults of middle age, the pressure drop is 2.5 mmHg and point 3 in children of age 8 years the pressure drop is 4.5 mmHg [26]. In a cohort of controls of average age 43 years old the normal arterial inflow was 792 mls/min [40] and in children of average age 8.3 years the inflow was 1200 ml/min [3]. Plotting the first two points as a straight line in Fig. 4 would give a pressure drop of 3.8 mmHg at 1200 ml/min but the known figure is 18% higher. As shown in Fig. 4 the simplest curve to link all three dots is a quadratic equation. Placing the mean arterial inflow for our controls of 990 ml/min in the formula returns a pressure drop across the venous system of 3.4 mmHg and by extrapolation the pressure drop for the mean blood flow in the hyperemic children of 1620 ml/min returns a pressure drop of 7.1 mmHg which is 3.7 mmHg higher. This is almost high enough to trigger a diagnosis of IIH in children who are not obese or sedated. Given the slight venous narrowing found in this cohort, the actual pressure drop is probably 1–2 mmHg higher than 3.7 mmHg. There is secondary imaging evidence of raised ICP in the hyperemic children, with 12 out of 19 or 63% having optic nerve sheaths greater than 6 mm (a 17% increase in optic nerve sheath diameter overall, Additional file 1: Table S2), 7 with globe flattening and 1 with a partly empty sella. Figure 5a is a box and whisker plot of the optic nerve sheath diameter for the controls, non-hyperemic patients at risk of IIH and hyperemic patients and suggests that an increase in blood flow increases ICP. If confirmed by follow-up studies, hyperemia should be considered for being included as a diagnostic criterion for childhood IIH.

One of the current authors (GB) developed a surrogate marker of raised cerebral sinus pressure which compares the cerebral arterial inflow at the skull base with the venous outflow of the major cerebral sinuses using MRI phase-contrast flow quantification. Initially this was applied to adults with normal pressure hydrocephalus [41] and later to adults with idiopathic and secondary intracranial hypertension [34]. It was found an elevation in venous pressure, from whatever cause, directed a larger percentage of the arterial inflow to exit via the smaller venous channels through the scalp, face and over the brain convexity through the veins of Trolard and Labbe as collateral flow. This collateral flow reduced the percentage of the arterial inflow returning via the main pathways (i.e. the straight and sagittal sinuses) [34]. This technique was subsequently applied to children with idiopathic hydrocephalus and an elevation in collateral flow was found to be similar to adults with IIH [42]. It later became apparent that there existed children with hydrocephalus who had arterial inflows greater than two standard deviations above the mean compared to controls in numbers greater than expected by chance alone [3, 8]. The hyperemic hydrocephalus patients again showed an increase in collateral flow bypassing the sagittal and straight sinuses, suggesting an increase in venous pressure [3]. The same protocol has now been extended to children referred for the indication “rule out idiopathic intracranial hypertension”. It is noted in the current cohort, the venous return percentage is reduced by 11% in the sagittal sinus and 4% in the straight sinus territory (p < 0.0001 and 0.0009) over all. Figure 5b is a box and whisker plot comparing the optic nerve sheath diameter in controls with the patients at risk of IIH who had a venous return both below and above 41%. Patients with a lower return percentage show optic nerve sheath dilatation and therefore a higher ICP. Given a reduction in the percentage venous return in the sagittal sinus two standard deviations from the mean is abnormal, and correlates with increased ICP, the cut-off for abnormal venous return would be 41%. In the entire cohort of patients, 14/42 or 33% would have abnormal venous return and 10/19 or 53% would be abnormal in the hyperemic patients, compared with none of the controls having abnormal venous return. This suggests there may be some utility in utilising this metric in children with the headache/IIH spectrum.

### Difference between IIH and hydrocephalus in children

In a companion publication outlining a 10 year review of childhood hydrocephalus [8], 56% had a high grade, venous outflow stenosis and 13% cerebral hyperemia [8]. In this cohort of patients with the headache/IIH continuum, one patient had a high grade stenosis (2%) and 45% had hyperemia, a significant difference. Rosman and Shands suggested, elevated venous pressure causes idiopathic intracranial pressure in adults (and older children) but causes hydrocephalus in younger children, the difference in outcome appears to depend on whether the cranial sutures are patent or closed [43]. In the previous companion study into childhood hydrocephalus, 37/55 or 67% children who presented with hydrocephalus were under 3 years of age [8] and presumably had open sutures allowing expansion of the cranium secondary to the elevated ICP increasing the CSF spaces. In the current study none of the children were under 3 years. The head circumference was only 3% larger than the controls suggesting the sutures were largely fused with no appreciable increase in head size. In addition, the 9% increase in brain volume found would account for the increase in intracranial volume from the 3% increase in circumference and would therefore exclude an increase in CSF volume developing within the ventricles. Interestingly, Alperin et.al. found an 8% increase in grey matter volume in adults with IIH [44] similar to the 9% increase in brain volume we found in children. They suggested the increased volume may be on the basis of an increase in interstitial fluid or blood [44]. In addition to age, in the children with hydrocephalus who were older than 4 years, the sagittal sinus was reduced in area by 35% [8]. In a recent study in an older hydrocephalus cohort, of mean age 44 years, there was an average 38% stenosis of the sagittal sinus by area, which generated an increase in the pressure gradient between the superficial and deep venous territories of 1.2 mmHg. This sagittal sinus narrowing was absent in adult IIH [45]. A narrowing of the sagittal sinus is also absent in the current cohort. Thus, a moderate sagittal sinus narrowing appears to correlate with ventricular enlargement in both adults and children and the lack of sagittal sinus narrowing with normal sized ventricles.

### Limitations, clinical perspective and future directions

One of the major limitations of this study is the practical difficulty in applying the existing gold standard in defining IIH in adults to children, with a disproportionate emphasis being placed on each of the criteria to ascertain a diagnosis of IIH as definite, probable and possible. The rationale of adapting criteria relevant for adults to the paediatric cohort is understandable given the CSF opening pressure by L.P is the only clinical tool available to assess intracranial pressure. Although the revised diagnostic criteria are written from the point of view that lumbar puncture is the gold standard [2], in practice many pediatric neurologists find L.P. far too invasive and the results if performed are either ambiguous or unhelpful in the clinical management, especially if the tap ends up dry or traumatic. The procedure is used sparingly and only in select difficult scenarios under a general anaesthetic. A spot L.P. measurement suffers from an inherent sampling error. A falsely reduced pressure, (taken at a single point in time) may be obtained at the nadir of the known pressure waves which occur in IIH, may be decreased by CSF leak from multiple attempts or by hyperventilation, conversely, a falsely elevated pressure can occur in a patient experiencing pain/ anxiety, performing a Valsalva or from poor positioning [46]. In one series utilising the response to treatment and the natural history of the patient’s condition as the gold standard in childhood IIH, the authors found that the initial L.P. had a false negative rate of 20% and a false positive rate of 14% [47] meaning one in three patients were misclassified. Lumbar puncture had an overall sensitivity of only 75%. We have used optic nerve sheath diameter as a surrogate for the L.P. Haredy et al. using direct ICP monitoring ± L.P. in a cohort of children measuring the ONSD from MRI images found a sensitivity of 71.4% and specificity of 89.7% when a threshold of 6 mm was used [20]. The minimum pressure change above baseline which can be resolved by the technique is estimated to be 5 mmHg [48]. These findings suggest that ONSD may actually perform better as a gold standard for IIH in children than a spot L.P. for a cross-sectional population study such as ours. However, this should be tempered by the limited resolution of the technique. Haredy et al. used a 1.5 T MRI, with 200 mm field of view and a 228 × 224 matrix giving an in-plane resolution of ± 0.9 mm. This is problematic given in their study, the average difference in diameter between the controls and patients with raised ICP was 1.14 mm. Our 3 T scanner has a higher inherent contrast resolution and utilised a 220 mm field of view with 256 × 256 matrix giving a marginally better in-plane resolution of 0.85 mm compared to Haredy et al., so we are confident the findings in our cohort are at least as accurate as the literature. For comparison a 7.5 MHz ultrasound probe has a lateral resolution of 0.6–0.7 mm [49] but poorer tissue contrast characterization than MRI. Given these findings, the resolution makes the interpretation of any one individual patient’s pressure less reliable compared to a larger cohort.

Given the difficulties with both L.P. and ONSD as gold standards, we would advocate a more pragmatic approach. The revised criteria cannot be used in the absence of an L.P. and the criteria for “suggested” IIH requires three out of four imaging criteria to be present however, these criteria are rarely found in children [47]. We failed to find one child who satisfied 3 out of 4 of these criteria. We would suggest in children who are thought on clinical grounds to be at high risk for IIH that three criteria are important, i.e. obesity adjusted for age, a transverse sinus stenosis greater than 65% by area and an arterial inflow greater than 1410 ml/min. If two or more of these criterial are present then the patient probably has IIH and should be treated appropriately. If they have only one criteria then L.P. may be helpful. If none of these three criteria are present, L.P. may be misleading. As a minimum, an MRI study comprising a 3DT1, axial T2, MR Venogram and arterial phase contrast flow quantification sequence should be performed. This takes about 20 min to acquire.

We can see two areas for further study. Given that the increase in arterial inflow we found is not due to a change in perfusion pressure, then the autoregulation of the CBF should be investigated for a change in signalling. Secondly, the conventional wisdom is that the venous outflow resistance is a constant and so pressure should be linearly related to flow (Ohm’s law). However, this appears to be wrong (see Fig. 4). One of our group (ARB) is currently completing a master’s thesis using computational fluid dynamics to find the cause of this apparent discrepancy.

## Conclusions

Cerebral hyperemia and mild venous outflow stenosis are associated with the headache/ IIH spectrum in children. Of these, hyperemia appears to be the most significant. A sagittal sinus return percentage less than 41% and an arterial inflow above 1410 ml/min may be useful markers of elevated venous and intracranial pressure.

## Supplementary information

**Additional file 1: Table S1.** Clinical findings of 42 patients with an indication of possible idiopathic intracranial hypertension (IIH). **Table S2.** Cerebral blood flow and optic nerve sheath size for control subjects and hyperemic and non- hyperemic groups, at risk for intracranial hypertension.

## Data Availability

All data generated or analysed during this study are included in this published article or additional files.

## Abbreviations

ALL: Acute lymphoblastic leukaemia
BMI: Body mass index
CBF: Cerebral blood flow
cmH_2_o: Centimeters of water
CNS: Central nervous system
CVP: Central venous pressure
D_e_: Effective diameter
FLAIR: Fluid attenuated inversion recovery
FR_csf_: CSF formation rate
Hd: Hydraulic diameter
ICP: Intracranial pressure
IIH: Idiopathic intracranial hypertension
IIHWOP: Idiopathic intracranial hypertension without papilledema
L.P.: Lumbar puncture
ml/100 g/min: Milliliters per 100 g per minute
mmHg: Millimeters of mercury
MRV: Magnetic resonance venography
R_out_: CSF outflow resistance
SSS_p_: Superior sagittal sinus pressure
TCBF: Total cerebral blood flow

## References

[1] Cleves-Bayon C (2018). Idiopathic intracranial hypertension in children and adolescents: an update. Headache.

[2] Friedman DI, Liu GT, Digre KB (2013). Revised diagnostic criteria for the pseudotumor cerebri syndrome in adults and children. Neurology.

[3] Bateman GA (2010). Hyperemic hydrocephalus: a new form of childhood hydrocephalus analogous to hyperemic intracranial hypertension in adults. J NeurosurgPediatr.

[4] Karimy JK, Duran D, Hu JK, Gavankar C, Gaillard JR, Bayri Y, Rice H, DiLuna ML, Gerzanich V, Marc Simard J, Kahle KT (2016). Cerebrospinal fluid hypersecretion in pediatric hydrocephalus. Neurosurg Focus.

[5] Blomquist HK, Sundin S, Ekstedt J (1986). Cerebrospinal fluid hydrodynamic studies in Children. J NeurolNeurosurgPsychiatr.

[6] Lalou AD, Czosnyk M, Czosnyka ZH, Krishnakumar D, Pickard JD, Higgins NJ (2020). Coupling of CSF and sagittal sinus pressure in adult patients with pseudotumour cerebri. Acta Neurochir.

[7] Guess HA, Charlton JD, Johnson RN, Mann JD (1985). A nonlinear least-squares method for determining cerebrospinal fluid formation and absorption kinetics in pseudotumor cerebri. Comput Biomed Res.

[8] Bateman GA, Yap SL, Subramanian GM, Bateman AR (2020). The incidence of significant venous sinus stenosis and cerebral hyperemia in childhood hydrocephalus: prognostic value with regards to differentiating active from compensated disease. Fluids Barriers CNS..

[9] Nadkarni T, Rekate HL, Wallace D (2004). Resolution of pseudotumor cerebri after bariatric surgery for related obesity. Case report. J Neurosurg..

[10] Cole TJ, Bellizzi MC, Flegal KM, Dietz WH (2000). Establishing a standard definition for child overweight and obesity worldwide: international survey. BMJ.

[11] Manjon JV, Coupe P. Volbrain an online MRI brain volumetry system. Front Neuroinform. 2016 http//doi.org**/**10.3389/fninf.2016.00030. ecollection. https://www.volbrain.ups.es. Accessed 21 Sept 2019. 10.3389/fninf.2016.00030 PMC4961698 27512372

[12] De Simone R, Ranieri A, Sansone M, Marano E, Valeria Russo C, Sacca F, Bonavita V (2019). Dural sinus collapsibility, idiopathic intracranial hypertension, and the pathogenesis of chronic migraine. Neurol Sci.

[13] Wang SJ, Silberstein SD, Patterson S, Young WB (1998). Idiopathic intracranial hypertension without papilledema: a case-control study in a headache centre. Neurology.

[14] Mathew NT, Ravishankar K, Sanin LC (1996). Coexistence of migraine and idiopathic intracranial hypertension without papilledema. Neurology.

[15] Dekaban AS (1978). Changes in brain weights during the span of human life: relation of brain weights to body heights and body weights. Ann Neurol.

[16] Gompertz RHC (1902). Specific gravity of the brain. J Physiol.

[17] Hirabuki N, Watanabe Y, Mano T, Fujita N, Tanaka H, Ueguchi T, Nakamura H (2000). Quantification of flow in the superior sagittal sinus performed with cine phase contrast MR imaging of healthy and chondroplastic children. AJNR Am J Neuroradiol.

[18] Chiron C, Raynauld C, Maziere B, Zilbovicius M, Laflamme L, Masure MC, Dulac O, Bourguignon M, Syrota A (1992). Changes in regional cerebral blood flow during brain maturation in children and adolescents. J Nucl Med.

[19] Arkuszewski M, Krejza J, Chen R, Melhem ER (2013). Sickle cell anemia: reference values of cerebral blood flow determined by continuous arterial spin labelling MRI. Neuroradiology.

[20] Haredy M, Zuccoli G, Tamber M, Davis A, Nischal K, Goldstein JA (2018). Use of neuroimaging measurements of optic nerve sheath diameter to assess intracranial pressure in craniostenosis. Child’s Nerv Sys.

[21] Karahalios DG, Rekate HL, Khayata MH, Apostolides PJ (1996). Elevated intracranial venous pressure as a universal mechanism in pseudotumor cerebri of varying etiologies. Neurology.

[22] Rowe FJ, Sarkies NJ (1999). The relationship between obesity and idiopathic intracranial hypertension. Int J ObesRelatMetab Discord.

[23] Sugerman HJ, DeMaria EJ, Felton WL, Nakatsuka M, Sismanis A (1997). Increased intra-abdominal pressure and cardiac filling pressures in obesity associated pseudotumor cerebri. Neurology.

[24] Sheldon CA, Paley GL, Xiao R, Kesler A, Eyal O, Ko MW, Boisvert CJ, Avery RA, Salpietro V, Phillips PH, Heidary G, McCormack SE, Liu GT (2016). Pediatric idiopathic intracranial hypertension: age, gender, and anthropometric features at diagnosis in a large. Retrospective, multisite cohort. Ophthalmology.

[25] Ahmed RM, Wilkinson M, Parker GD, Thurtell MJ, Macdonald J, McCluskey PJ, Allan R, Dunne V, Hanlon M, Owler BK, Halmagyi GM (2011). Transverse sinus stenting for idiopathic intracranial hypertension: a review of 52 patients and of model predictions. AJNR Am J Neuroradiol.

[26] Bateman GA, Siddique SH (2014). Cerebrospinal fluid absorption block at the vertex in chronic hydrocephalus: obstructed arachnoid granulations or elevated venous pressure?. Fluids Barriers CNS..

[27] Barbagallo M, Vitaliti G, Greco F, Pavone P, Matin N, Panta G, Lubrano R, Falsaperla R (2017). Idiopathic intracranial hypertension in a paediatric population: a retrospective observational study on epidemiology, symptoms and treatment. J Biol RegulHomeost Agents.

[28] Dwyer CM, Prelog K, Owler BK (2013). The role of venous sinus outflow obstruction in pediatric idiopathic intracranial hypertension. J NeurosurgPediatr.

[29] Enzmann DR, Pelc NJ (1992). Cerebrospinal fluid flow measured by phase-contrast cine MR. AJNR Am J Neuroradiol.

[30] Evans AJ, Iwai F, Grist TA, Sostman HD, Hedlund LW, Spritzer CE (1993). Magnetic resonance imaging of blood flow with a phase subtraction technique. In vitro and in vivo validation. Invest Radio.

[31] Laffon E, Valli N, Latrabe V, Franconi JM, Barat JL, Laurent F (1998). A validation of a flow quantification by MR phase mapping software. Eur J Radiol.

[32] Powell AJ, Maier SE, Chung T, Geva T (2000). Phase-velocity cine magnetic resonance imaging measurement of pulsatile blood flow in children and young adults: in vitro and in vivo validation. PediatrCardiol.

[33] Youngson RM (2005). Collins dictionary of medicine.

[34] Bateman GA (2002). Vascular hydraulics associated with idiopathic and secondary intracranial hypertension. AJNR Am J Neuroradiol.

[35] Bateman GA, Stevens SA, Stimpson J (2009). A mathematical model of idiopathic intracranial hypertension incorporating increased arterial inflow and variable venous outflow collapsibility. J Neurosurg.

[36] Brian JE (1998). Carbon dioxide and the cerebral circulation. Anaesthesiology.

[37] Symon L (1970). Regional cerebrovascular responses to acute ischaemia in normocapnia and hypercapnia. J NeurolNeurosurgPsychiatr.

[38] Shawcross DL, Davies NA, Mookerjee RP, Hayes PC, Williams R, Lee A, Jalan R (2004). Worsening of cerebral hyperemia by the administration of Terlipressin in acute liver failure with severe encephalopathy. Hepatology.

[39] Avery RA, Shah SS, Licht DJ, Seiden A, Huh JW, Boswinkel J (2010). Reference range for cerebrospinal fluid opening pressure in children. N Eng J Med.

[40] Bateman GA, Lechner-Scott J, Lea RA (2016). A comparison between the pathophysiology of multiple sclerosis and normal pressure hydrocephalus: is pulse wave encephalopathy a component of MS?. Fluids Barriers CNS.

[41] Bateman GA (2000). Vascular compliance in normal pressure hydrocephalus. AJNR Am J Neuroradiol.

[42] Bateman GA, Smith RL, Siddique SH (2007). Idiopathic hydrocephalus in children and idiopathic intracranial hypertension in adults: two manifestations of the same pathophysiological process?. J Neurosurg.

[43] Rosman NP, Shands KN (1978). Hydrocephalus caused by increased intracranial pressure: a clinicopathological study. Ann Neurol.

[44] Alperin N, Ranganathan S, Bagci AM, Adams DJ, Ertl-Wagner B, Saraf-Lavi E, Skylar EM, Lam BL (2013). Evidence of impaired CSF homeostasis in obesity-associated idiopathic intracranial hypertension. AJNR Am J Neuroradiol.

[45] Bateman GA, Bateman AR (2019). Differences in the calculated transvenous pressure drop between chronic hydrocephalus and idiopathic intracranial hypertension. AJNR Am J Neuroradiol.

[46] Mallery RM, Rehmani OF, Woo H, Chen YJ, Reddi S, Salman KL (2019). Utility of magnetic resonance imaging features for improving the diagnosis of idiopathic intracranial hypertension without papilledema. J Neuro-Opthalmol.

[47] Inger HE, Rogers DL, McGregor ML, Aylward SC, Reem RE. Diagnostic criteria in pediatric hypertension. J AAPOS. 2017;21:492–495.e.2 10.1016/j.jaapos.2017.08.003 29081363

[48] Hansen H, Lagreze W, Kreuger O, Helmke K (2011). Dependence of the optic nerve sheath diameter on acutely applied subarachnoid pressure- an experimental ultrasound study. Acta Ophthalmol.

[49] Lochner P, Behnke S, Fassbender K, Andrejewski A, Knodel S, Siniscalchi A (2018). Simulation and experimental characterization of lateral imaging resolution of ultrasound systems and assessment of system suitability for acoustic optic nerve sheath diameter measurement. J Neuroimaging..

