# Supplementary material for: The incidence of obesity, venous sinus stenosis and cerebral hyperaemia in children referred for MRI to rule out idiopathic intracranial hypertension at a tertiary referral hospital: a 10 year review
Source: Fluids Barriers CNS. 2020 Sep 29;17:59. doi: 10.1186/s12987-020-00221-4 (PMC7526159; doi:10.1186/s12987-020-00221-4)
Supplement: Supplementary file 1 — Additional file 1: Table S1. Clinical findings of 42 patients with an indication of possible idiopathic intracranial hypertension (IIH). Table S2. Cerebral blood flow and optic nerve sheath size for control subjects and hyperemic and non- hyperemic groups, at risk for intracranial hypertension. [file 12987_2020_221_MOESM1_ESM.docx]

**Table S1.** Clinical findings of 42 patients with an indication of possible idiopathic intracranial hypertension (IIH)

| **Case** | **Age**  **yrs** | **Sex** | **Clinical** | **Body habitus** | **Head circumference**  **cm** | **Revised diagnostic criteria** |
| --- | --- | --- | --- | --- | --- | --- |
| 1 | 4.8 | male | migraine vomiting ataxia | obese | 52.6 | No IIH |
| 2 | 8.1 | male | Headache papilledema | normal | 52.5 | No IIH |
| 3 | 3.3 | male | migraine with aura | obese | 52.0 | No IIH |
| 4 | 8.8 | female | headache | normal | 50.6 | No IIH |
| 5 | 5.1 | female | photophobia behavioral disturbance | obese | 56.4 | No IIH |
| 6 | 9.8 | male | headache | normal | 51.4 | No IIH |
| 7 | 4.3 | male | atrioseptal defect headache | normal | 52.4 | No IIH |
| 8 | 14.4 | female | headache, blurred vision, papilledema, L.P. 35 cmH2O | obese | 58.6 | Definite IIH |
| 9 | 10.2 | male | headache | normal | 57.4 | No IIH |
| 10 | 11.5 | male | migraine papilledema | normal | 55.4 | No IIH |
| 11 | 12.7 | female | IIH, vision loss, papilledema, L.P. 30 cmH2O | obese | 55.3 | Definite IIH |
| 12 | 10.8 | male | headache nystagmus vomiting | normal | 57.6 | No IIH |
| 13 | 11.5 | female | headache papilledema | obese | 57.6 | No IIH |
| 14 | 3.8 | female | headache | obese | 50.1 | No IIH |
| 15 | 4.7 | female | headache | normal | 52.0 | No IIH |
| 16 | 7.9 | female | Chiari 1 headache | normal | 52.5 | No IIH |
| 17 | 5.5 | female | incidental papilledema at optometrist | overweight | 55.0 | No IIH |
| 18 | 7.8 | female | KIF1a related disorder headache | normal | 48.5 | No IIH |
| 19 | 12.9 | female | headache papilledema | normal | 55.1 | No IIH |
| 20 | 12.6 | male | ALL remission headache L.P. 24 cmH2O | overweight | 55.4 | No IIH |
| 21 | 3.4 | male | headache papilledema | normal | 50.6 | No IIH |
| 22 | 5.8 | male | headache | normal | 50.9 | No IIH |
| 23 | 6.2 | male | ALL remission headache papilledema L.P. 26cmH2O under general anesthetic | normal | 50.3 | Probable IIH |
| 24 | 10.9 | male | headache | obese | 55.3 | No IIH |
| 25 | 13.7 | male | migraine | normal | 55.1 | No IIH |
| 26 | 13.8 | female | Turners syndrome growth hormone headache | overweight | 54.3 | No IIH |
| 27 | 10.5 | female | chronic ear infections migraine | normal | 52.3 | No IIH |
| 28 | 7.8 | male | headache | overweight | 50.9 | No IIH |
| 29 | 11.2 | female | headache | overweight | 53.4 | No IIH |
| 30 | 7.1 | female | epilepsy chronic headache | overweight | 50.4 | No IIH |
| 31 | 15.0 | female | complex regional pain syndrome headache L.P. 17 cmH2O | obese | 60.7 | No IIH |
| 32 | 8.7 | female | incidental papilledema at optometrist | normal | 52.1 | No IIH |
| 33 | 12.7 | male | migraine | overweight | 56.8 | No IIH |
| 34 | 12.9 | female | epilepsy headache | normal | 54.1 | No IIH |
| 35 | 10.2 | female | headache visual loss | normal | 54.4 | No IIH |
| 36 | 5.9 | female | headache | normal | 56.1 | No IIH |
| 37 | 13.9 | male | headache drusen | normal | 52.6 | No IIH |
| 38 | 12.1 | female | headache | normal | 53.7 | No IIH |
| 39 | 15.0 | female | headache | normal | 54.3 | No IIH |
| 40 | 12.2 | male | migraine | normal | 52.1 | No IIH |
| 41 | 9.5 | male | acute post viral headache papilledema | obese | 54.8 | No IIH |
| 42 | 8.1 | male | neonatal stroke headache | normal | 48.9 | No IIH |
|  |  |  |  |  |  |  |
| Mean | 9.5 |  |  |  | 53.6 |  |
| SD | 3.5 |  |  |  | 2.7 |  |

Note.- ALL, acute lymphoblastic leukaemia; cm, centimeters; cmH2O, cm of water; IIH, idiopathic intracranial hypertension; L.P., lumbar puncture; yrs, years.

**Table S2.** Cerebral blood flow and optic nerve sheath size for control subjects and hyperemic and non- hyperemic groups, at risk for intracranial hypertension.

|  |  |  | |  | **Control** |  |  |  | |  | |  |
| --- | --- | --- | --- | --- | --- | --- | --- | --- | --- | --- | --- | --- |
| **Case** | **Age**  **years** | **Arterial inflow**  **ml/min** | | **Sagittal sinus flow**  **ml/min** | **Straight**  **sinus flow**  **ml/min** | **SSS %**  **flow** | **ST %**  **Flow** | **Brain volume**  **cm^3^** | | **CBF**  **ml/100cm^3^/min** | | **Average ONSD**  **mm** |
| 1 | 7.2 | | 1250 | 780 | 240 | 62 | 19 | 1744 | 72 | | 5.7 | |
| 2 | 10.3 | | 1310 | 790 | 200 | 60 | 15 | 1453 | 90 | | 4.0 | |
| 3 | 5.7 | | 940 | 540 | 170 | 57 | 18 | 1250 | 75 | | 5.2 | |
| 4 | 15.0 | | 810 | 520 | 80 | 64 | 10 | 919 | 88 | | 4.8 | |
| 5 | 7.2 | | 1250 | 780 | 240 | 62 | 19 | 1148 | 109 | | 5.2 | |
| 6 | 8.4 | | 1400 | 660 | 180 | 47 | 13 | 1244 | 112 | | 6.2 | |
| 7 | 13.4 | | 1050 | 630 | 70 | 60 | 7 | 1210 | 87 | | 5.5 | |
| 8 | 6.4 | | 990 | 590 | 220 | 59 | 22 | 1255 | 78 | | 4.9 | |
| 9 | 15.0 | | 690 | 330 | 80 | 48 | 12 | 1377 | 52 | | 6.1 | |
| 10 | 13.0 | | 890 | 440 | 110 | 49 | 12 | 1199 | 74 | | 5.4 | |
| 11 | 9.6 | | 830 | 480 | 160 | 58 | 19 | 1022 | 81 | | 5.7 | |
| 12 | 5.5 | | 850 | 440 | 120 | 52 | 14 | 1090 | 78 | | 5.1 | |
| 13 | 7.1 | | 1100 | 560 | 160 | 50 | 14 | 1025 | 107 | | 4.8 | |
| 14 | 3.0 | | 750 | 560 | 190 | 75 | 25 | 1160 | 64 | | 6.2 | |
| 15 | 4.8 | | 830 | 570 | 140 | 69 | 16 | 1143 | 72 | | 5.5 | |
| 16 | 2.6 | | 1342 | 780 | 310 | 58 | 23 | 1765 | 76 | | 5.7 | |
| 17 | 5.5 | | 770 | 520 | 150 | 68 | 20 | 1157 | 66 | | 4.9 | |
| 18 | 13.8 | | 800 | 360 | 140 | 45 | 17 | 950 | 84 | | 4.8 | |
| 19 | 12.6 | | 1000 | 500 | 160 | 50 | 16 | 1307 | 77 | | 5.5 | |
| 20 | 9.6 | | 860 | 500 | 80 | 58 | 10 | 964 | 90 | | 4.5 | |
| 21 | 11.5 | | 1230 | 560 | 360 | 45 | 30 | 1310 | 94 | | 4.8 | |
| 22 | 14.3 | | 940 | 440 | 160 | 47 | 17 | 1297 | 72 | | 6.1 | |
|  |  | |  |  |  |  |  |  |  | |  | |
| mean | 9.2 | | 990 | 560 | 170 | 57 | 17 | 1224 | 82 | | 5.3 | |
| SD | 4.1 | | 210 | 130 | 70 | 8 | 5 | 219 | 15 | | 0.6 | |
|  |  | |  |  |  |  |  |  |  | |  | |
|  |  | |  |  | **At risk for IIH** **Hyperemic** |  |  |  |  | |  | |
| **Case** |  | | **Arterial inflow**  **ml/min** | **Sagittal sinus flow**  **ml/min** | **Straight sinus flow**  **ml/min** | **SSS % flow** | **ST%**  **Flow** | **Brain volume**  **cm^3^** | **CBF**  **ml/100cm^3^/min** | | **Average ONSD**  **mm** | |
| 1 | 4.8 | | 2030 | 820 | 300 | 40 | 15 | 1357 | 150 | | 6.8 | |
| 2 | 8.1 | | 1960 | 770 | 190 | 39 | 10 | 1383 | 142 | | 5.7 | |
| 3 | 3.3 | | 1790 | 800 | 300 | 45 | 17 | 1392 | 128 | | 7.3 | |
| 4 | 8.8 | | 1730 | 530 | 180 | 31 | 10 | 1234 | 140 | | 4.7 | |
| 5 | 5.1 | | 1670 | 920 | 230 | 55 | 14 | 1630 | 103 | | 7.1 | |
| 6 | 9.8 | | 1660 | 660 | 140 | 40 | 8 | 1291 | 129 | | 5.8 | |
| 7 | 4.3 | | 1640 | 800 | 240 | 49 | 15 | 1401 | 117 | | 5.4 | |
| 8 | 14.4 | | 1610 | 410 | 120 | 25 | 7 | 1376 | 117 | | 6.9 | |
| 9 | 10.2 | | 1610 | 750 | 130 | 46 | 8 | 1464 | 110 | | 4.9 | |
| 10 | 11.5 | | 1600 | 590 | 180 | 37 | 11 | 1447 | 111 | | 7.0 | |
| 11 | 12.7 | | 1560 | 430 | 140 | 28 | 9 | 1099 | 142 | | 7.5 | |
| 12 | 10.8 | | 1530 | 730 | 200 | 48 | 13 | 1460 | 105 | | 5.0 | |
| 13 | 11.5 | | 1500 | 500 | 120 | 34 | 8 | 1412 | 106 | | 6.4 | |
| 14 | 3.8 | | 1490 | 630 | 260 | 43 | 18 | 1095 | 136 | | 5.2 | |
| 15 | 4.7 | | 1460 | 770 | 180 | 52 | 12 | 1241 | 118 | | 6.6 | |
| 16 | 7.9 | | 1460 | 840 | 210 | 58 | 14 | 1343 | 109 | | 6.8 | |
| 17 | 5.5 | | 1450 | 810 | 150 | 56 | 10 | 1560 | 93 | | 6.7 | |
| 18 | 7.8 | | 1440 | 500 | 220 | 34 | 15 | 1252 | 115 | | 6.7 | |
| 19 | 12.9 | | 1430 | 540 | 170 | 38 | 12 | 1383 | 103 | | 6.2 | |
|  |  | |  |  |  |  |  |  |  | |  | |
| **Hyperemic mean** | 8.3 | | 1620 | 670 | 190 | 42 | 12 | 1358 | 120 | | 6.2 | |
| **Hyperemic SD** | 3.5 | | 190 | 150 | 60 | 10 | 3 | 1136 | 16 | | 0.9 | |
| **t-test** | 0.48 | | <0.0001* | 0.01* | 0.26 | <0.0001* | 0.001* | 0.03* | <0.0001* | | 0.0002* | |
|  |  | |  |  | **At risk for IIH** **non Hyperemic** |  |  |  |  | |  | |
| 20 | 12.6 | | 1400 | 650 | 210 | 46 | 15 | 1470 | 95 | | 5.8 | |
| 21 | 3.4 | | 1380 | 670 | 230 | 49 | 16 | 1302 | 106 | | 6.6 | |
| 22 | 5.8 | | 1370 | 540 | 70 | 39 | 5 | 1253 | 111 | | 5.6 | |
| 23 | 6.2 | | 1370 | 780 | 330 | 57 | 24 | 1264 | 108 | | 5.5 | |
| 24 | 10.9 | | 1320 | 490 | 100 | 37 | 8 | 1472 | 90 | | 5.2 | |
| 25 | 13.7 | | 1230 | 690 | 110 | 56 | 9 | 1407 | 88 | | 5.6 | |
| 26 | 13.8 | | 1210 | 600 | 120 | 50 | 10 | 1338 | 91 | | 4.9 | |
| 27 | 10.5 | | 1150 | 500 | 90 | 43 | 8 | 1210 | 95 | | 3.8 | |
| 28 | 7.8 | | 1140 | 430 | 200 | 37 | 18 | 1122 | 101 | | 5.7 | |
| 29 | 11.2 | | 1130 | 670 | 120 | 60 | 11 | 1394 | 81 | | 4.4 | |
| 30 | 7.1 | | 1100 | 560 | 120 | 50 | 11 | 1025 | 107 | | 5.1 | |
| 31 | 15.0 | | 1100 | 440 | 90 | 40 | 8 | 1606 | 68 | | 5.1 | |
| 32 | 8.7 | | 1080 | 510 | 190 | 47 | 18 | 1409 | 77 | | 4.9 | |
| 33 | 12.7 | | 1070 | 530 | 150 | 50 | 14 | 1634 | 66 | | 5.3 | |
| 34 | 12.9 | | 1010 | 640 | 180 | 63 | 18 | 1208 | 84 | | 3.7 | |
| 35 | 10.2 | | 1000 | 440 | 130 | 44 | 13 | 1323 | 76 | | 5.1 | |
| 36 | 5.9 | | 970 | 520 | 100 | 53 | 11 | 1309 | 74 | | 4.6 | |
| 37 | 13.9 | | 970 | 410 | 200 | 42 | 21 | 1347 | 72 | | 5.6 | |
| 38 | 12.1 | | 950 | 440 | 140 | 47 | 15 | 1164 | 82 | | 4.5 | |
| 39 | 15.0 | | 950 | 440 | 120 | 47 | 13 | 1207 | 78 | | 4.6 | |
| 40 | 12.2 | | 850 | 390 | 90 | 46 | 11 | 1305 | 65 | | 5.7 | |
| 41 | 9.5 | | 790 | 450 | 110 | 57 | 14 | 1365 | 58 | | 4.4 | |
| 42 | 8.1 | | 710 | 390 | 110 | 55 | 15 | 1078 | 66 | | 4.9 | |
|  |  | |  |  |  |  |  |  |  | |  | |
| **Non hyperemic mean** | 10.5 | | 1010 | 530 | 140 | 48 | 13 | 1321 | 84 | | 5.1 | |
| **Non hyperemic SD** | 3.4 | | 190 | 110 | 60 | 7 | 5 | 152 | 16 | | 0.7 | |
| **t-test** | 0.25 | | 0.09 | 0.41 | 0.23 | 0.001* | 0.02* | 0.12 | 0.58 | | 0.26 | |
| **All cohort mean** | 9.5 | | 1330 | 600 | 170 | 46 | 13 | 1334 | 100 | | 5.6 | |
| **All cohort**  **SD** | 3.5 | | 310 | 150 | 60 | 10 | 4 | 145 | 24 | | 1.0 | |
| **t-test** | 0.73 | | <0.0001* | 0.34 | 0.88 | <0.0001* | 0.0009* | 0.02* | 0.005* | | 0.19 | |

Note.- cm^3^, centimeter cubed; SSS, superior sagittal sinus; ST, straight sinus; ml/100cm^3^/min, milliliters per 100 centimeters cubed per minute; mm, millimeters; mm^2^, millimeters squared; SD, standard deviation; ONSD, optic nerve sheath diameter; ^*^ , t-test p value < 0.05. All patient groups were compared to the control group in the t-tests.
